# Researching Mental Health Disorders in the Era of Social Media: Systematic Review

**DOI:** 10.2196/jmir.7215

**Published:** 2017-06-29

**Authors:** Akkapon Wongkoblap, Miguel A Vadillo, Vasa Curcin

**Affiliations:** ^1^ Department of Informatics King's College London London United Kingdom; ^2^ Primary Care and Public Health Sciences King’s College London London United Kingdom; ^3^ Departamento de Psicología Básica Universidad Autónoma de Madrid Madrid Spain

**Keywords:** mental health, mental disorders, social networking, artificial intelligence, machine learning, public health informatics, depression, anxiety, infodemiology

## Abstract

**Background:**

Mental illness is quickly becoming one of the most prevalent public health problems worldwide. Social network platforms, where users can express their emotions, feelings, and thoughts, are a valuable source of data for researching mental health, and techniques based on machine learning are increasingly used for this purpose.

**Objective:**

The objective of this review was to explore the scope and limits of cutting-edge techniques that researchers are using for predictive analytics in mental health and to review associated issues, such as ethical concerns, in this area of research.

**Methods:**

We performed a systematic literature review in March 2017, using keywords to search articles on data mining of social network data in the context of common mental health disorders, published between 2010 and March 8, 2017 in medical and computer science journals.

**Results:**

The initial search returned a total of 5386 articles. Following a careful analysis of the titles, abstracts, and main texts, we selected 48 articles for review. We coded the articles according to key characteristics, techniques used for data collection, data preprocessing, feature extraction, feature selection, model construction, and model verification. The most common analytical method was text analysis, with several studies using different flavors of image analysis and social interaction graph analysis.

**Conclusions:**

Despite an increasing number of studies investigating mental health issues using social network data, some common problems persist. Assembling large, high-quality datasets of social media users with mental disorder is problematic, not only due to biases associated with the collection methods, but also with regard to managing consent and selecting appropriate analytics techniques.

## Introduction

Mental illness is quickly becoming one of the most serious and prevalent public health problems worldwide [1]. Around 25% of the population of the United Kingdom have mental disorders every year [2]. According to statistics published by the World Health Organization, more than 350 million people have depression. In terms of economic impact, the global costs of mental health problems were approximately US $2.5 trillion in 2010. By 2030, it is estimated that the costs will increase further to US $6.0 trillion [3]. Mental disorders include many different illnesses, with depression being the most prominent. Additionally, depression and anxiety disorders can lead to suicidal ideation and suicide attempts [1]. These figures show that mental health problems have effects across society, and demand new prevention and intervention strategies. Early detection of mental illness is an essential step in applying these strategies, with the mental illnesses typically being diagnosed using validated questionnaires designed to detect specific patterns of feelings or social interaction [4-6].

Online social media have become increasingly popular over the last few years as a means of sharing different types of user-generated or user-curated content, such as publishing personal status updates, uploading pictures, and sharing current geographical locations. Users can also interact with other users by commenting on their posts and establishing conversations. Through these interactions, users can express their feelings and thoughts, and report on their daily activities [7], creating a wealth of useful information about their social behaviors [8]. To name just 2 particularly popular social networks, Facebook is accessed regularly by more than 1.7 billion monthly active users [9] and Twitter has over 310 million active accounts [10], producing large volumes of data that could be mined, subject to ethical constraints, to find meaningful patterns in users’ behaviors.

The field of data science has emerged as a way of addressing the growing scale of data, and the analytics and computational power it requires. Machine learning techniques that allow researchers to extract information from complex datasets have been repurposed to this new environment and used to interpret data and create predictive models in various domains, such as finance [11], economics [12], politics [13], and crime [14]. In medical research, data science approaches have allowed researchers to mine large health care datasets to detect patterns and accrue meaningful knowledge [15-18]. A specific segment of this work has focused on analyzing and detecting symptoms of mental disorders through status updates in social networking websites [19].

Based on the symptoms and indicators of mental disorders, it is possible to use data mining and machine learning techniques to develop automatic detection systems for mental health problems. Unusual actions and uncommon patterns of interaction expressed in social network platforms [19] can be detected through existing tools, based on text mining, social network analysis, and image analysis.

Even though the current performance of predictive models is suboptimal, reliable predictive models will eventually allow early detection and pave the way for health interventions in the forms of promoting relevant health services or delivering useful health information links. By harnessing the capabilities offered to commercial entities on social networks, there is a potential to deliver real health benefits to users.

This systematic review aimed to explore the scope and limits of cutting-edge techniques for predictive analytics in mental health. Specifically, in this review we tried to answer the following questions: (1) What methods are researchers using to collect data from online social network sites such as Facebook and Twitter? (2) What are the state-of-the-art techniques in predictive analytics of social network data in mental health? (3) What are the main ethical concerns in this area of research?

## Methods

We conducted a systematic review to examine how social media data have been used to classify and predict the mental health state of users. The procedure followed the guidelines of the Preferred Reporting Items for Systematic Reviews and Meta-Analyses (PRISMA) to outline and assess relevant articles [20].

### Literature Search Strategy

We searched the literature in March 2017, collecting articles published between 2010 and March 8, 2017 in medical and computer science databases. We searched PubMed, Institute of Electrical and Electronics Engineers (IEEE Xplore), Association for Computing Machinery (ACM Digital Library), Web of Science, and Scopus using sets of keywords focused on the prediction of mental health problems based on data from social media. We restricted our searches to common mental health disorders, as defined by the UK National Institute for Health and Care Excellence [21]: depression, generalized anxiety disorder, panic disorder, phobias, social anxiety disorder, obsessive-compulsive disorder (OCD), and posttraumatic stress disorder (PTSD). To ensure that our literature search strategy was as inclusive as possible, we explored Medical Subject Headings (MeSH) for relevant key terms. MeSH terms were used in all databases that made this option available. Search terms are outlined in Textbox 1.

In addition, we manually searched the proceedings of the Computational Linguistics and Clinical Psychology Workshops (CLPsych) and the outputs of the World Well-Being Project [22] to find additional articles that our search terms might have excluded. Furthermore, we examined the reference lists of included articles for additional sources.

**Search strategy to identify articles on the prediction of mental health problems based social media data.**

Medical Subject Headings (MeSH)

Depression/ or Mental Health/ or Mental Disorders/ or Suicide or Life Satisfaction/ or Well Being/ or Anxiety/ or Panic/ or Phobia/ or OCD/ or PTSD
Social Media/ or Social Networks/ or Facebook/ or Twitter/ or Tweet
Machine Learning/ or Data Mining/ or Big Data/ or Text Analysis/ or Text Mining/ or Predictive Analytics/ or Prediction/ or Detection/ or Deep Learning
(1) and (2)
(1) and (3)

### Inclusion and Exclusion Criteria

We further filtered the titles and abstracts of articles retrieved using the search terms outlined in Textbox 1. Only articles published in peer-reviewed journals and written in English were included. Further inclusion criteria were that studies had to (1) focus on predicting mental health problems through social media data, and (2) investigate prediction or classification models based on users’ text posts, network interactions, or other features of social network platforms. Within this review, we focused on social network platforms—that is, those allowing users to create personal profiles, post content, and establish new or maintain existing relationships.

Studies were excluded if they (1) only analyzed the correlation between social network data and symptoms of mental illness, (2) analyzed textual contents only by human coding or manual annotation, (3) examined data from online communities (eg, LiveJournal), (4) focused on the relationship between social media use and mental health disorders (eg, so-called Internet addiction), (5) examined the influence of cyberbullying on mental health, or (6) did not explain where the datasets came from.

### Data Extraction

After screening articles and obtaining a set of studies that met our inclusion criteria, we extracted the most relevant data from the main texts. These are title, author, aims, findings, methods, data collection on machine learning techniques, sampling, questionnaire, platform, and language.

## Results

### Overview

**Figure 1:**
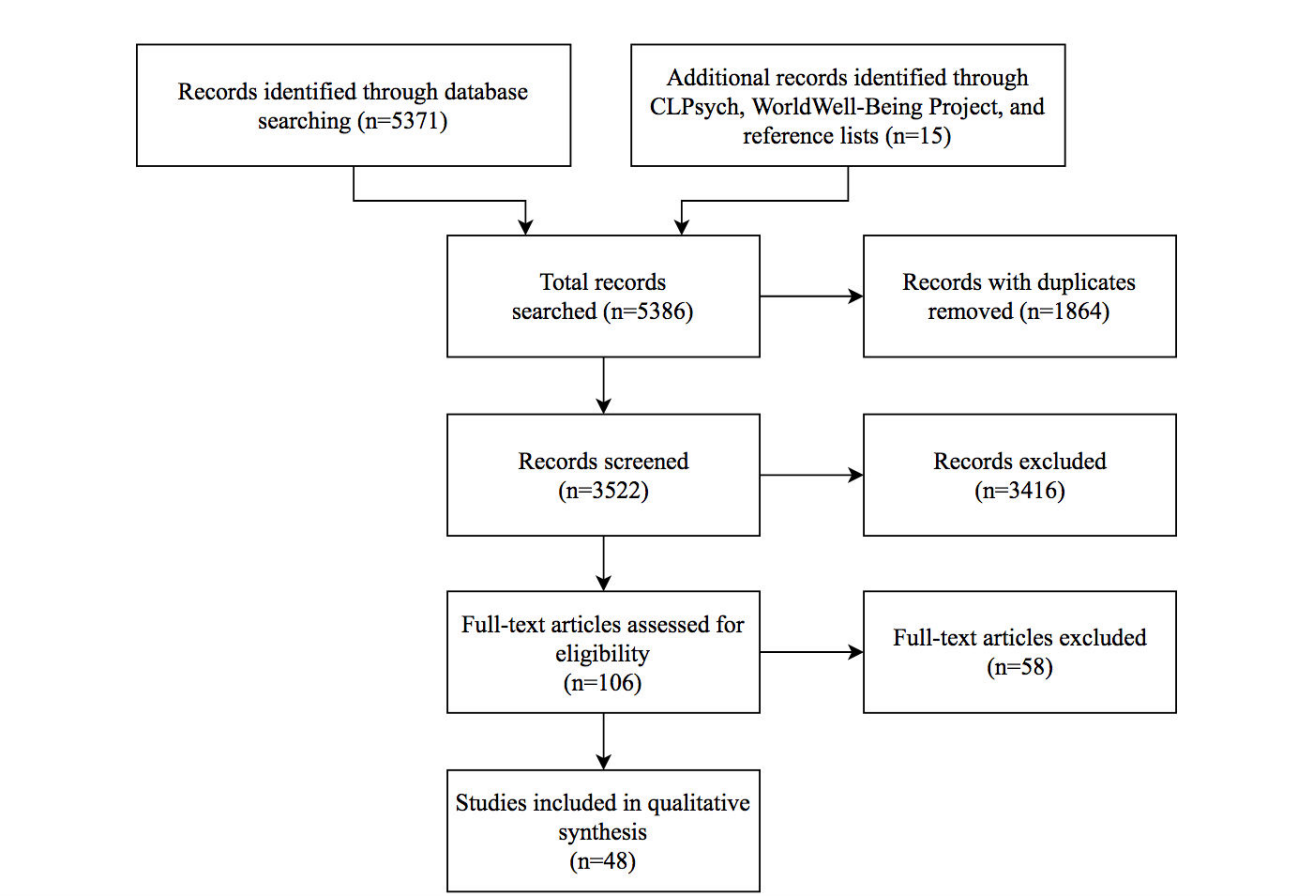
Preferred Reporting Items for Systematic Reviews and Meta-Analyses (PRISMA) flow diagram. CLPsych: Computational Linguistics and Clinical Psychology Workshops.

Figure 1 presents a PRISMA flow diagram of the results of searching and screening articles following the above search methodology. The initial search resulted in a total of 5371 articles plus 11 additional articles obtained through CLPsych, 1 from the World Well-Being Project, and 3 from the reference lists of included articles. We removed 1864 of these articles because of duplication. Each of the remaining articles (n=3522) was screened by reviewing its title and abstract. If an article analyzed data from other sources (such as brain signals, mental health detection from face detection, or mobile sensing), we discarded it. This resulted in a set of 106 articles. By matching these with our inclusion and exclusion criteria, we removed a further 58 articles. To sum up, we excluded 5338 articles and included 48 in the review (see Figure 1).

We extracted data from each of the 48 articles. Table 1 and Multimedia Appendix 1 (whose format is adapted from previous work [11,23]) show the key characteristics of the selected studies [24-71], ordered by year published. Of the studies reviewed, 46 were published from 2013 onward, while only 2 peer-reviewed articles were published between 2011 and 2012. None of the selected articles was published in 2010.

**Table 1:** Summaries of articles reviewed.

First author, date, reference	Aims	Findings
Wang, 2017 [24]	To explore and characterize the structure of the community of people with eating disorders using Twitter data and then classify users into those with and without the disorder.	There was assortativity among users with eating disorder. The classifier distinguished 2 groups of people.
Volkava, 2016 [25]	To explore academic discourse from tweets and build predictive models to analyze the data.	Tweets from students across 44 universities were related to student surveys on satisfaction and happiness.
Saravia, 2016 [26]	To present a new data collection method and classify individuals with mental illness and nonmental illness.	The proposed method and a classifier were built as an online system, which distinguished 2 groups of individuals and provided mental illness information.
Kang, 2016 [27]	To propose classification models to detect tweets of users with depression for a long period of time. Classifiers were based on the texts, emoticons, and images they posted.	The models detected users with depression.
Schwartz, 2016 [28]	To present predictive models to estimate individual well-being through textual content on social networks.	A combination of message- and user-level aggregation of posts performed well.
Chancellor, 2016 [29]	To explore posts from Instagram to forecast levels of mental illness severity of pro-eating disorder.	Future mental illness severity could be predicted from user-generated messages.
Braithwaite, 2016 [30]	To explore machine learning algorithms to measure suicide risk in the United States.	Machine learning algorithms successfully classified users with suicidal ideation.
Coppersmith, 2016 [31]	To explore linguistics and emotional patterns in Twitter users with and without suicide attempt.	There were quantifiable signals of suicide attempt in tweets.
Lv, 2015 [32]	To build a Chinese suicide dictionary, based on Weibo posts, to detect suicide risk.	The Chinese suicide dictionary detected individuals and tweets at suicide risk.
O’Dea, 2015 [33]	To explore machine learning models to automatically detect the level of concern for each suicide-related tweet.	Machine learning classifiers estimated the level of concern from suicide-related tweets.
Liu, 2015 [34]	To investigate and predict users’ subjective well-being based on Facebook posts.	Users’ subjective well-being could be predicted from posts and their time frame.
Burnap, 2015 [35]	To explore suicide-related tweets to understand users’ communications on social media.	Classification models classified tweets into relevant suicide categories.
Park, 2015 [36]	To analyze the relationships between Facebook activities and the depression state of users.	Participants with depression had fewer interactions, such as receiving likes and comments. Depressed users posted at a higher rate.
Hu, 2015 [37]	To present classifiers with different lengths of observation time to detect depressed users.	Behavioral and linguistic features predicted depression. A 2-month period of observation enabled prediction cues of depression half a month in advance.
Tsugawa, 2015 [38]	To develop a model to recognize individuals with depression from non-English social media posts and activities.	Activities extracted from Twitter were useful to detect depression; 2 months of observation data enabled detection of symptoms of depression. The topics estimated by LDA^a^ were useful.
Zhang, 2015 [39]	To explore 2 natural language processing algorithms to identify posts predicting the probability of suicide.	LDA automatically detected suicide probability from textual contents on social media.
Coppersmith, 2015 [40]	To explore tweet content with self-reported health sentences and language differences in 10 mental health conditions.	There were quantifiable signals of 10 mental health conditions in social network messages and relations between them.
Preotiuc-Pietro, 2015 [41]	To implement linear classifiers to detect users with PTSD^c^ and depression based on user metadata, and several textual and topic features.	The combination of linear classifiers performed better than average classifiers. All unigram features performed well.
Mitchell, 2015 [42]	To use several natural language processing techniques to explore the language of schizophrenic users on Twitter.	Character ngram features were used to train models to classify users with and without schizophrenia. LDA outperformed linguistic inquiry and word count.
Preotiuc-Pietro, 2015 [43]	To study differences in language use in tweets about mental health depending on the role of personality, age, and sex of users.	Personality and demographic data extracted from tweets detected users with depression or PTSD.
Pedersen, 2015 [44]	To explore and study the accuracy of decision lists of ngrams to classify users with depression and PTSD.	Bigram features underperformed ngram 1-6 features.
Resnik, 2015 [45]	To build classifiers to categorize depressed and nondepressed users, based on supervised topic models.	LDA mined useful information from tweets. Supervised topic models such as supervised LDA and supervised anchor model improved LDA accuracy.
Resnik, 2015 [46]	To build classifiers with TF-IDF^d^ weighting, using support vector machine with a linear kernel or radial basis function kernel.	TF-IDF showed good performance, and TF-IDF with supervised topic model performed even better.
Durahim, 2015 [47]	To explore data from social networks to measure the Gross National Happiness of Turkey.	Sentiment analysis estimated Gross National Happiness levels similar to Turkish statistics.
Guan, 2015 [48]	To explore 2 types of classifiers to detect posts revealing high suicide risk.	Users’ profiles and their generated text were used to classify users with high or low suicide risk.
Landeiro Dos Reis, 2015 [49]	To explore exercise-related tweets to measure their association with mental health.	Users who posted workouts regularly tended to express lower levels of depression and anxiety.
De Choudhury, 2014 [50]	To explore several types of Facebook data to detect and predict postpartum depression.	Postpartum depression was predicted from an increase of social isolation and a decrease of social capital.
Huang, 2014 [51]	To present a framework to detect posts related to suicidal ideation.	The best predictive model was based on support vector machine.
Wilson, 2014 [52]	To explore the types of mental health information posted and shared on Twitter.	The study distinguished 8 themes of information about depression in Twitter posts, each having different features.
Coppersmith, 2014 [53]	To present a novel method to collect posts related to PTSD and build a classifier.	The classifier distinguished users with and without self-reported PTSD.
Kuang, 2014 [54]	To create the Chinese version of the extended PERMA^e^ corpus and use it to measure happiness scores.	The proposed model measured happiness.
Hao, 2014 [55]	To propose machine learning models to measure subjective well-being of social media users.	The model measured subjective well-being from social media data.
Prieto, 2014 [56]	To develop a machine learning model to detect and measure the prevalence of health conditions.	The proposed methods identified the presence of health conditions on Twitter.
Lin, 2014 [57]	To develop a deep neural network model to classify users with or without stress.	The trained model detected stress from user-generated content.
Schwartz, 2014 [58]	To build predictive models to detect depression based on Facebook text.	Facebook updates enabled distinguishing depressed users. Predictive models offered insights into seasonal affective disorder.
Coppersmith, 2014 [59]	To analyze tweets related to health and propose a new method to quickly collect public tweets containing statements of mental illnesses.	There were differences in quantifiable linguistic signals of bipolar disorder, depression, PTSD, and seasonal affective disorder in tweets.
Homan, 2014 [60]	To examine the potential of tweet content to classify suicidal risk factors.	Annotations from novices and experts were used to train classifiers, although expert annotations outperformed novice annotations.
Park, 2013 [61]	To develop a Web app to detect symptoms of depression from features extracted from Facebook.	Depressed users had fewer Facebook friends, used fewer location tags, and tended to have fewer interactions.
Wang, 2013 [62]	To build a depression detection model based on sentiment analysis of data from social media.	Sentiment analysis with 10 features detected users with depression, with 80% accuracy.
Wang, 2013 [63]	To explore a detection model, based on node and linkage features, to recognize the presence of depression in social media users. This was an extended version of their earlier study [62].	The node and linkage features model performed better than the model based just on node features.
Tsugawa, 2013 [64]	To explore the effectiveness of an analytic model to estimate depressive tendencies from users’ activities on a social network.	There was a correlation between the Zung Self-Rating Depression Scale and the model estimations.
De Choudhury, 2013 [65]	To explore predictive models to classify mothers with a tendency to change behavior after giving birth or to experience postpartum depression.	Tweets during prenatal and early postnatal periods predicted future behavior changes, with an accuracy of 71%. Data over 2-3 weeks after giving birth improved prediction results, with an accuracy of 80%-83%.
De Choudhury, 2013 [66]	To explore the potential of a machine learning model to measure levels of depression in populations.	The proposed model estimated levels of depression.
De Choudhury, 2013 [67]	To develop a prediction model to classify individual users with depression.	The predictive model classified users with depression.
Schwartz, 2013 [68]	To analyze tweets from different US counties to predict well-being of people in those areas.	Topic features provided useful information about life satisfaction.
Hao, 2013 [69]	To explore the mental state of users through their online behavior.	Online behavior enabled prediction of mental health problems.
Jamison-Powell, 2012 [70]	To explore the characteristics of tweets that included the #insomnia hashtag.	Tweets about insomnia contained more negative words. People used Twitter to express their symptoms and ideas for coping strategies.
Bollen, 2011 [71]	To explore an online social network to measure subjective well-being levels of users and calculated assortativity.	There was assortativity among Twitter users.

^a^LDA: latent Dirichlet allocation.

^b^CLPsych: Computational Linguistics and Clinical Psychology Workshops.

^c^PTSD: posttraumatic stress disorder.

^d^TF-IDF: term frequency-inverse document frequency.

^e^PERMA: positive emotions, engagement, relationships, meaning, and accomplishment.

The selected studies can be divided into several distinct categories. Several studies [27,36-38,40-46,52,56-59,61-64, 66,67] used datasets from social networks to examine depression. Postpartum depression disorder was explored by De Choudhury et al [50,65], PTSD was investigated by 8 studies [40,41,43-46,53,59]. Anxiety and OCD were investigated by 2 studies [40,69]. Borderline disorder and bipolar disorder were investigated by 3 studies [26,40,59]. Seasonal affective disorder was studied by Coppersmith et al [40,59]. Eating disorder was explored by Chancellor et al [29], Coppersmith et al [40], and Prieto et al [56]. Attention-deficit/ hyperactivity disorder, anxiety, and schizophrenia were examined by Coppersmith et al [40], and sleep disorder was studied by Jamison-Powell et al [70]. None of the included studies explored phobias or panic disorders. Users with suicidal ideation were investigated by 8 studies [30-33,35,39,51,60]. Happiness, satisfaction with life, and well-being were investigated by 7 studies [28,34,47,54, 55,68,71].

Of the studies included in this review, 31 analyzed social network contents written in English [24-31,33-35,40-46,49, 50,52,53,58-60,65-68,70,71]; 11 studies investigated Chinese text [32,37,39,48,51,54,55,57,62,63,69]; 2 focused on Korean [36,61] and 2 on Japanese text [38,64], 1 looked at Turkish content [47], and 1 jointly at Spanish and Portuguese [56].

### Data Collection Techniques

Each of the selected articles was based on a dataset directly or indirectly obtained from social networks. We identified 2 broad approaches to data collection: (1) collecting data directly from the participants with their consent using surveys and electronic data collection instruments (eg, Facebook apps), and (2) aggregating data extracted from public posts.

The methods for collecting data directly from participants varied with the purpose of the studies and the target platform. These methods included posting project information on relevant websites inviting participants to take part in the project [32,38,50] and posting tasks on crowdsourcing platforms asking for project volunteers [28,30,66,67]. For crowdsourcing, researchers posted detailed information about their studies on platforms such as Amazon Mechanical Turk [74] to attract participants. As part of a questionnaire, the participants would typically be asked to provide informed consent allowing collection of their social network data.

A range of questionnaires were used to measure participants’ levels of depression and life satisfaction, including the Center for Epidemiologic Studies Depression Scale [36,38,61,66,67], Patient Health Questionnaire-9 [50], Beck Depression Inventory [36,38,61,67], Zung Self-Rating Depression Scale [64], Depressive Symptom Inventory-Suicidality Subscale [30], and Symptom Checklist-90-Revised [69]. The instruments used to detect suicidal ideation and the possibility of an individual committing suicide were the Suicide Probability Scale [32,39,48], the Acquired Capability for Suicide Scale [30], and the Interpersonal Needs Questionnaire [30]. Satisfaction with life and well-being were measured with the Satisfaction with Life Scale [28,34], the Positive and Negative Affect Schedule [55], and the Psychological Well-Being Scale [55]. One study used the Revised NEO Personality Inventory-Revised to assess personality [58].

The second approach was to pool only public posts from social network platforms, by using regular expressions to search for relevant posts, such as “I was diagnosed with [condition name]” [40,42,43,59].

To collect social network data, each data source required a custom capture mechanism, due to a lack of standards for data collection. Facebook-based experiments gathered user datasets by developing custom tools or Web apps connecting to the Facebook application programming interfaces (APIs) [36,50,61]. Another group of studies used Twitter APIs to explore cues for mental disorders [24-27,30,31,33,35,38,47,52,53,56-60,64-68, 70,71]. A similar approach was used for Instagram APIs [29] and Sina Weibo APIs [32,37,39,51,54,55,57,62,63].

Another way of obtaining data was promoted by the myPersonality project, which provides both social network data and a variety of psychometric test scores for academic researchers [75], and was used by 3 studies [28,34,58]. Some studies [41,44-46] originated from workshops where the organizers shared data already approved by an institutional review board (IRB) for analysis.

### Translating Collected Data Into Knowledge and Results

In all of the selected studies, several standard steps had to be taken before machine learning algorithms could be applied to data. First, data were cleaned and preprocessed to ensure that they were in the form required by the analytical algorithms. Second, the key features (the term “feature” in machine learning denotes a set of observations that is relevant to the modelling problem, typically represented numerically [76]) related to the research domain were prepared for model construction. Overall, this involves feature extraction and feature selection, producing sets of features to be used in learning and validating predictive models.

#### Data Preprocessing

The corpus of data is typically preprocessed by (1) removing unsuitable samples and (2) cleaning and preparing the data for analysis. Information and questionnaires from participants might contain useless data and incomplete details, which are usually removed from studies in order to improve the accuracy of prediction and classification results. Participants who take an abnormally short or long time to complete the questionnaires were excluded from 4 studies [38,39,66,67]. Low-activity participants who had published less than a defined threshold of posts were removed from 8 studies [26,32,34,37,39,55,59,71]. Participants with poor correlations between 2 different questionnaires were excluded from the final dataset in 2 studies [38,67].

As part of the data cleaning process, each post was checked for the majority written language (eg, contained at least 70% English words [28,40,42,53,59,70]). This ensured that the available tools were suitable to analyze the posts. Each post was preprocessed by eliminating stop words and irrelevant data (eg, retweets, hashtags, URLs), lowercasing characters, and segmenting sentences [31,44,46,53,56,60,66]. Emoticons were converted to other forms such as ASCII codes [45] to ensure data were machine readable. Anonymization was also performed to remove any potentially identifiable usernames [31,33,35,52,53,70].

#### Feature Extraction

There are many potential techniques to extract features that could be used for predicting mental health problems in social network users. Several studies have attempted to investigate the textual contents of social networks to understand what factors contain cues for mental disorders. However, some research projects have used alternative techniques. In this review, we identified three broad approaches to feature extraction: text analysis, image analysis, and social interaction.

In text mining, sentiment analysis is a popular tool for understanding emotion expression. It is employed to classify the polarity of a given text into categories such as positive, negative, and neutral [77]. Several studies [24,28,30,32,34,39, 49,50,52-55,57,60,65-68,70] used the well-known linguistic inquiry and word count (LIWC) [78] to extract potential signals of mental problems from textual content (eg, the word frequency of the first personal pronoun “I” or “me” or of the second personal pronoun, positive and negative emotions being used by a user or in a post). OpinionFinder [79] was used by Bollen et al [71] and SentiStrength [80] was used by Kang et al [27] and by Durahim and Coşkun [47] to carry out sentiment analysis. Custom tools were also developed for performing sentiment analysis. Affective Norms for English Words [81] was used to qualify the emotional intensity of English words in 2 studies [65,66], while topic modelling was employed in 4 studies [28,29,38,39] to extract topics from user-generated posts.

Social media posts tend to be rich in various emoticons. As a consequence, several studies [27,62] looked into the meaning and mood states associated with their use.

Apart from posting text messages, social network platforms allow users to post images. Some studies investigated these images for cues of mental disorders [27,57]. Color compositions and scale-variant feature transform descriptor techniques were used to extract emotional meanings of each individual image [27]. Image properties, comprising color theme, saturation, brightness, color temperature, and color clarity, were analyzed be Lin et al [57].

Finally, social network platforms contain millions of interactions and relationships among users. Social network users not only can connect and add online friends, but also can post, comment, and reply to their friends. The resulting graph structure, comprising information about interactions, relationships, and friendships, was mined to understand the cues that can be connected to symptoms of mental disorders (eg, interactions among depressed users and assortative mixing patterns) [24,63,71].

#### Feature Selection

Feature selection isolates a relevant subset of features that are able to predict symptoms of mental disorders or correctly label participants, while avoiding overfitting. Statistical analysis is typically performed to discover a set of parameters that can differentiate between users with mental disorders and users without mental disorders. The techniques used in the selected studies were Pearson correlation coefficient [36,55,56], correlation-based feature selection [56], Spearman rank correlation coefficient [61], and Mann-Whitney *U* test [61]. The dimensionality of features was reduced by principal component analysis [35,58,65-67], randomized principal component analysis [28], convolutional neural network with cross-autoencoder technique [57], forward greedy stepwise [37], binary logistic regression [62], gain ratio technique [56], and relief technique [56].

#### Predictive Model Construction

In the selected studies, prediction models were used to detect and classify users according to mental disorders and satisfaction with life. To build a predictive model, a selected set of features is used as training data for machine learning algorithms to learn patterns from those data.

All the articles included in this review used supervised learning techniques, where the sample data contain both the inputs and the labeled outputs. The model learns from these to predict unlabeled inputs from other sources and provide prediction outputs [82]. The techniques used in these studies included support vector machine (SVM) [32,33,35,38,42,56,69], linear SVM [24,27,41,46,60], and SVM with a radial basis function kernel [24,27,46,51,65-67]. Regression techniques included ridge regression [28], linear regression [37,58], log-linear regression [53,59], logistic regression [25,31,33,37,48,49,51], binary logistic regression with elastic net regularization [41,43], linear regression with stepwise selection [39,55,64], stepwise logistic regression with forward selection [50], regularized multinomial logistic regression [29], linear support vector regression [45,55], least absolute shrinkage and selection operator [55,68], and multivariate adaptive regression splines [55]. Other algorithms used for binary classification were decision trees [35,51,56,62,63], random forest [26,48,51], rules decision [62], naive Bayes [24,35,51,56,62,69], k-nearest neighbor [24,56], maximum entropy [42], neural network [69], and deep learning neural network [57].

#### Model Verification

Following model construction, its accuracy was measured using a test dataset. The most common model validation technique was *n*-fold cross-validation, which randomly partitions a dataset into *n* equal subsets and proceeds to iterate *n* times, with each subset used for validation exactly once, while the remaining *n–* 1 subsets are used as training data [82]. Several studies [26,31,35,37-43,49,51,56,67,69] employed 10-fold cross-validation to verify their prediction models and classifiers, while 5-fold cross-validation was used by 4 studies [24,48,55,57]. Leave-one-out cross-validation was used in 2 studies [30,59].

The performance of predictive models can also be evaluated in other datasets. Several studies [27-29,33,45,46,49,58,60,64,68] divided the collected dataset into training and test subsets to measure the accuracy of their models. Some [47,48,53,54,66] collected a new dataset to evaluate the accuracy of the predicted results and compare the predicted results with a set of known statistics (eg, depression rates in US cities, student satisfaction survey, and Gross National Happiness percentages of provinces of Turkey).

### Ethics

The ethical aspects of using social network data for research are still not clearly defined, particularly when working with information that is publicly available. Thus, the studies that we surveyed adopted a wide range of approaches to handle ethical constraints.

Among the articles included in this review, 9 [30,32,33,36,38,40,42,48,61] were approved by their authors’ IRBs, and 8 [34,36-38,48,50,55,61] reported receiving informed consent from participants prior to data analysis. For public data collected from crowdsourcing platforms, participants who opted in provided their consent to data sharing [67]. For myPersonality data, Liu et al [34] stated that the dataset itself had IRB approval, so the authors did not report obtaining any further approval from their institution. Youyou et al [83] also concluded that no IRB approval was needed for using myPersonality data. Chancellor et al [29] did not seek IRB approval, because their study used Instagram data without personally identifiable information.

Researchers in 6 studies [31,33,35,52,53,70] reported that the social network datasets collected from participants were anonymized. O’Dea et al [33] removed names, user identifiers, and user identities, and the data collected had to be analyzed after 3 months. Names and usernames in tweets were removed or replaced with other text in 3 studies [31,52,53]. Jamison-Powell et al [70] reported that they removed user identifiers from tweets illustrated in their published article.

The performance of these models is still fuzzy and unstable. As a consequence, none of these studies presented the model’s predicted output to participants themselves. Schwartz et al [28] also noted that mental health predictive models are still under development and not sufficiently accurate to be used in practice, and little research has been done on user acceptability of such tools.

## Discussion

### Principal Findings

The purpose of this review was to investigate the state of development of research on machine learning techniques predicting mental health from social network data. This review also focused on identifying gaps in research and potential applications to detect users with mental health problems. Despite the thousands of articles collected through our search terms, the results of our review suggest that there is a relatively small but growing number of studies using machine learning models to predict mental health problems from social network data. From the initial set of matched articles, only 48 met our inclusion criteria and were selected for review. Some of the excluded studies focused on analysis of the effects of social media use on mental health and well-being states of individual users, and the influence of cyberbullying in social networks on other users.

### What Were the Most Surprising Findings?

From the above results, we observed that the same methods could be adapted to analyze posts in different languages. For example, Tsugawa et al [38] adapted De Choudhury’s methods [67], originally designed for the analysis of English textual content, to Japanese textual content. Both of them achieved similar results, although some outcomes were dissimilar due to differences in contexts and cultures. This example illustrates that same methods can be used to facilitate studies in different languages.

Several sites were used as sources of data. Facebook is possibly the most popular social network platform. However, only a few studies relied on Facebook datasets to predict mental disorders. One reason for this might be that, by default, users on this site do not make their profiles publicly accessible. Another reason is that getting data from Facebook requires consent from users.

From the selected studies, we can acknowledge several benefits and drawbacks of the methods used in the experiments.

#### Data Collection

Twitter was a popular source of social network data in the surveyed articles. It provides two different ways of accessing the data: retrospective (using their search APIs) and prospective (via their streaming APIs). Retrospective access allows a regular expression search on the full set of historical tweets, while prospective access allows a search to be set to capture all matching tweets going forward. However, the prospective search grants access to a sample of only 1% of all real-time public tweets based on specific filters. Twitter provides an alternative resource, Firehose, which can provide a standing search over all public tweets, as used in some studies [65-68], but it is only accessible through paid subscription [84].

There are some important differences between studies conducted on Facebook and those using microblogging platforms like Twitter or Sina Weibo. Facebook does not allow developers to access interactions and friendships between users. In addition, users must provide explicit consent to allow an app to pool their data. As a consequence of this, no previous research has used social network analysis to measure and predict mental health problems from Facebook data. On the other hand, microblogging sites grant access to such data. These sites provide APIs that allow developers to get information about followers and followees, and to construct social network graphs of interacting users.

In terms of data collection from users, there are some differences between obtaining data through participants’ consent and using regular expression to search for relevant posts. The former option can provide us the real results of the prevalence of mental disorders from participants. The latter approach reduces the time and cost of identifying users with mental illness [59].

#### Feature Extraction Techniques

The LIWC tool is mostly used for text analysis in psychological research. It extracts many category features, such as style words, emotional words, and parts of speech, from textual contents. It is relatively easy to use and does not require programming skills. Users can just select and open a file or a set of files and LIWC will extract the relevant features and values of each feature. However, there are some disadvantages too. First, LIWC is a proprietary software and users have to purchase a license to use it. Second, the feature database of the tool is not easy to modify. To do this, researchers might need programming skills.

To overcome these shortcomings, there are alternative tools to extract features. However, these tools are rather limited in that they can extract only some features. WordNet is a large English lexicon that can be used to extract parts of speech from text and find semantic meanings of words [85]. SentiStrength assesses the polarity between positive and negative words and the levels of strength of positive and negative words in a textual message [80]. OpinionFinder performs subjectivity, objectivity, and sentiment analysis [79]. Mallet is a useful natural language processing tool to classify or cluster documents, create topics, and perform sequence labelling [86]. Latent Dirichlet allocation is a useful and powerful technique to create topic models. Latent Dirichlet allocation analyzes latent topics, based on word distribution, and then assigns a topic to each document [87]. Each word from an assigned text can be tagged with parts of speech by Part-Of-Speech Tagger [88].

### What Can Be Done to Improve the Area?

The selected articles were largely focused on depression, at around 46% (22/48), while 17% (8/48) focused on suicide. Nearly 15% (7/48) of the articles reported a study of well-being and happiness. The rest of the articles investigated postpartum depression, eating disorder, and PTSD. Worthy of note, there is a lack of models for detection of chronic stress and anxiety disorders. Only 1 study in our sample built a stress state detection model [57]. Therefore, this is likely to become an interesting avenue for future research. If a user has a long period of chronic stress, he or she might be becoming depressed. For instance, Hammen [89] reported that chronic stress is a symptomatic source of depression and can develop into other disorders.

Furthermore, based on the selected articles, no study used social network data from actual patients with mental illnesses clinically identified by a doctor or psychologist. Most of the studies included in our review assessed mental disorders with surveys, which are open to self-identifying biases. It would be interesting to promote a closer collaboration between computer scientists and doctors or psychologists, who could provide access to patients with a diagnosis of mental disorders. This might improve the accuracy and reliability of data, making it possible to build predictive models based on features extracted from real patients’ social networks. However, mental health conditions might only be formally diagnosed in a specific subset of patients with those conditions, which may lead to a different type of bias.

Importantly, this area of research can benefit from the adoption of open science standards [90]. Many of the studies we reviewed were based on an analysis of openly available data from social networking sites or from the myPersonality project. Parts of the materials used in some of the studies are posted online (eg, [28,32]) or available upon request (eg, [61]). However, most of the studies did not share their entire computational workflow, including not only the datasets, but also the specific code used to preprocess and analyze them. Therefore, future studies should comply with the Transparency and Openness Promotion guidelines [91] at level 2 (which requires authors to deposit data and code in trusted repositories) or 3 (which also requires the reported analyses to be reproduced independently before publication). Of course, to avoid the dissemination of sensitive personal information, the datasets should be properly deidentified when necessary.

### What Are the Novel Trends?

The next generation of predictive models will include more technical analyses. Most of the selected studies relied on textual analysis. But apart from text mining techniques, other methods can be used to gain insights into mental disorders in collected datasets. For instance, image analysis can be used to extract meaningful features from images posted by users. Users facing mental disorders may post images with specific color filters or contents. Among our reviewed studies, 2 found a significant relationship between emotions and color use [92,93]. Another interesting technique is social network analysis. In this review, we selected 3 studies that used social network analysis to examine mental health. However, only 2 studies analyzed symptoms of mental disorders through social network analysis [24,63], while 1 study explored well-being [71]. One study reported that symptoms of depression can be observed through social networks. In other words, depression can be detected through each person’s friends [94]. These examples show that social network analysis is a promising tool to investigate the prevalence of mental illness among online users.

A wide range of machine learning algorithms were used in the reviewed studies. Only 1 study used deep learning algorithms to build a classifier [57], with the rest relying on SVMs, regression models, and decision trees to build classification models. It is expected that, with the rise in popularity of deep learning techniques, this will be changing soon. However, deep learning models are a black box, as opposed to human-interpretable models, such as regression and decision trees, raising the issue of whether it is possible, or indeed necessary, to have these algorithms validated by clinical experts [95].

**Figure 2:**
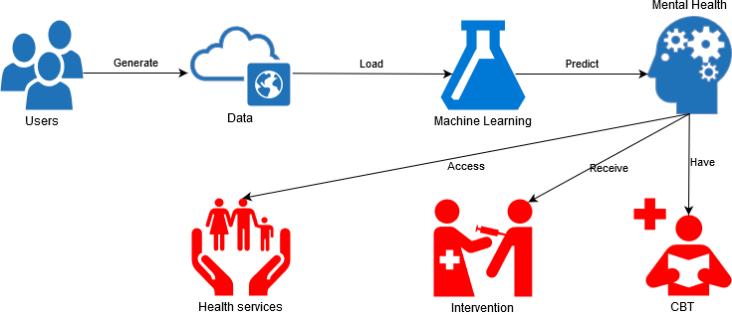
Conceptual view of social network-based mental health research. CBT: cognitive behavioral therapy.

As this review showed, it is now possible to detect social network users with mental health problems. However, a supporting methodology needs to be developed to translate this innovation into practice and provide help to individuals. Thus, mechanisms are needed to integrate the data science efforts with digital interventions on social network platforms, such as promoting access to health services, offering real-time interventions [96], delivering useful health information links, and conducting cognitive behavioral therapy [97] (see Figure 2).

### Ethical Concerns

Several studies outside the scope of this review are particularly useful in highlighting the importance of ethical issues in this area of research. For instance, researchers from Facebook and Cornell University [98] collected and used datasets from Facebook, without offering the possibility to opt out. According to the US Federal Policy for the Protection of Human Subjects (‘Common Rule’), all studies conducted in the United States are required to offer an opt-out for participants. However, private companies do not fall under this rule [99]. This study was not approved by the Cornell University IRB either, “[b]ecause this experiment was conducted by Facebook, Inc. for internal purposes, the Cornell University IRB determined that the project did not fall under Cornell’s Human Research Protection Program” [99].

Another study collected public Facebook posts and made the dataset publicly available to other researchers on the Internet [100]. The posts were manually collected by accessing authors’ friends’ profiles, and anonymizing them. But even so, the posts could still be easily identified [101].

As a result of privacy issues in research with human subjects, the Association of Internet Researchers and other authors have proposed not only ethical questions to evaluate the ethical implications of a research project before starting, but also specific guidelines to eliminate and deal with these issues [102,103].

Surprisingly, few of the studies focused on ethical issues. Conway [104] provided a taxonomy of ethical concepts to bear in mind when using Twitter data for public health studies. Conway [104] and McKee [105] reviewed and presented normative rules for using public Twitter data, including paraphrasing collected posts, receiving informed consent from participants, hiding a participant’s identity, and protecting collected data. Some ethical issues, including context sensitivity, complication of ethics and methodology, and legitimacy requirements, were explicitly addressed by Vayena et al [106].

Mikal et al [107] focused on the perspectives of participants in using social media for population health monitoring. The authors reported that most research participants agreed to have their public posts used for health monitoring, with anonymized data, although they also thought that informed consent would be necessary in some cases.

One approach to reducing the ethical issues of accessing to and using personal information in this area of research is to anonymize the collected datasets to prevent the identification of participants. Wilkinson et al [103] suggested that researchers should not directly quote messages or the public URLs of messages in publication, because these can be used to identify content creators. Sula [108] provided strategies to deal with research in social media including involving participants in studies (not just collect public contents), not collect personally identifiable information (eg, social network profile names), provide participants with a chance to opt out, and make resulting research findings easily accessible and understandable to participants. In most localities, doing any research that collects private information (including social networking posts) from human participants is required to provide project information to IRBs or ethics committees to obtain approval prior to data collection [102,109].

### Related Work

This review focused on studies building predictive machine learning models to automatically detect mental health conditions from social network data. Some studies linking mental health and other sources of data did not meet our selection criteria but provide interesting insights about research trends in this area. For instance, previous research has tried to predict mental health conditions or suicidal risk from alternative sources of data such as clinical notes [110], voice analysis [111,112], face analysis [113], and multimodal analysis [114]. We excluded other studies from this review because they used social media data to predict different outcomes; for example, Hanson et al [115] used Twitter data to predict drug abuse. Additionally, recent work has investigated reasons behind Twitter users posting about their mental health [116].

### Conclusion

The purpose of this review was to provide an overview of the state-of-the-art in research on machine learning techniques predicting mental health from social network data. Most of the selected studies approached this problem using text analysis. However, some studies also relied on image analysis and social network analysis to gain insights into mental health problems from social network datasets. Predictive models and binary classifiers can be trained based on features obtained from all these techniques. Based on our selected articles, there were relatively few studies applying predictive machine learning models to detect users with mental disorders in real social networks. Moving forward, this research can help in designing and validating new classification models for detecting social network users with mental illnesses and recommend a suitable individually tailored intervention. These interventions might be delivered in the form of advertisements, information links, online advice, or cognitive behavioral therapy; for example, Facebook is considering offering users deemed at risk of suicide online help in real time [117]. However, the reliability of the provided social network data and the general desirability of such interventions should be carefully studied with the users.

With advances in smart data capture devices, such as mobile phones, smart watches, and fitness accessories, future research could combine physical symptoms, such as movements, heart signs, or sleep patterns, with online social network activity to improve the accuracy and reliability of predictions. Finally, scholars interested in conducting research in this area should pay particular attention to the ethical issues of research with human subjects and data privacy in social media, as these are still not fully understood by ethics boards and the wider public.

## Abbreviations

API: application programming interface
CLPsych: Computational Linguistics and Clinical Psychology Workshops
IRB: institutional review board
LIWC: linguistic inquiry and word count.
MeSH: Medical Subject Headings
OCD: obsessive-compulsive disorder
PRISMA: Preferred Reporting Items for Systematic Reviews and Meta-Analyses
PTSD: posttraumatic stress disorder
SVM: support vector machine

## Appendix

Multimedia Appendix 1 Summaries of methodologies of the selected articles.
