# Supplementary material for: Researching Mental Health Disorders in the Era of Social Media: Systematic Review
Source: J Med Internet Res. 2017 Jun 29;19(6):e228. doi: 10.2196/jmir.7215 (PMC5509952; doi:10.2196/jmir.7215)
Supplement: Multimedia Appendix 1 [file jmir_v19i6e228_app1.pdf]

First author, date, reference	Methodology	Machine language techniques	Data collection	Sampling	Survey	Site	Language of the study
Wang, 2017 [24]	Key features were extracted from Twitter data. Classifiers were trained.	LIWC[a] extracted 80 features. Naive Bayes, SVM[b] with linear, RBF[c], sigmoid, and polynomial kernels and k-nearest neighbor were used to train models.	Datasets were collected from Twitter by searching user profiles with eating disorder keywords.	3380 users with eating disorder and a random sample of 68,667 users	None	TW[d]	English

First author, date, reference	Methodology	Machine language techniques	Data collection	Sampling	Survey	Site	Language of the study
Volkava, 2016 [25]	Researchers manually categorized tweets with course names into 3 academic disclosure types. Features were extracted. Predictive models were trained with the disclosure labels. The prediction models, sentiment classifier, and emotion classifier analyzed tweets with university names.	Ngram, TF-IDF[e], GLoVe[f], normalized pointwise mutual information, and Word2vec were used to extract features. Logistic regression was used to train predictive models.	Tweets were searched with university and course names.	79,329 tweets mentioning universities, and 2074 tweets mentioning course names	None	TW	English

First author, date, reference	Methodology	Machine language techniques	Data collection	Sampling	Survey	Site	Language of the study
Saravia, 2016 [26]	Features were extracted. A classifier was trained, and then the trained model was used to develop an online classification system.	TF-IDF was used to weight the importance of target words in a document. Sentiment140 API[g] extracted the polarity of each tweet. Random forest was used to train a classifier.	Datasets were manually collected from users, who provided profile descriptions of their mental illness, in health communities on Twitter.	481 users with mental illness and a random sample of 548 users	None	TW	English

First author, date, reference	Methodology	Machine language techniques	Data collection	Sampling	Survey	Site	Language of the study
Kang, 2016 [27]	A total of 3 models were developed to define a mood label for each given text, emoticon, and image. A prediction model using the 3 models was developed to identify users with depression moods. The model was tested with the available datasets. Tweets were collected by search APIs, and then used to test the prediction model.	Visual sentiment ontology and SentiStrength were used as sentiment corpora. WordNet identified parts of speech. SVM with an RBF kernel was used to build image classifiers. Linear SVM was used to build a text classifier.	Datasets were searched from Twitter with “kill myself” as the keyword and 10 people names from The New York Times.	23,956 tweets from 35 users with depression and 10 users without depression	None	TW	English

First author, date, reference	Methodology	Machine language techniques	Data collection	Sampling	Survey	Site	Language of the study
Schwartz, 2016 [28]	A crowdsourcing task was posted on Mechanical Turk to code PERMA[h] [72] and SWLS[i] scores of 5100 status updates, and then the coded posts were downloaded. Prediction models (message-level, user-level, and cascaded message-to-user-level well-being prediction models) were developed. The 2 datasets were used to train and test the models.	Ngrams were employed to extract use of an informal text tokenizer. LDA[j] was used to obtain topics. LIWC estimated positive and negative words. Randomized [PCAk] reduced dimensionality. Ridge regression was used to build a prediction model.	Messages were downloaded from the myPersonality project.	260,840 messages from 2198 users	SWLS PERMA	FB[l]	English

First author, date, reference	Methodology	Machine language techniques	Data collection	Sampling	Survey	Site	Language of the study
Chancellor, 2016 [29]	A topic model was built from posts. Researchers coded the severity of each topic. An automated mental illness severity rater was developed based on the coded topics. The rater was then evaluated. Predictive models for mental illness severity were built.	LDA was used to find topics and topic probability distribution. Regularized multinomial logistic regression was used to build predictive models.	The Instagram API collected data by initial keywords. The obtained dataset was used to expand the set of keywords, which was used to search for users with eating disorder.	26 million public posts related to eating disorders from 100,000 users	None	IG[m]	English
Braithwaite, 2016 [30]	Key features were extracted from tweets. A classifier was implemented based on LIWC features and a class label.	LIWC extracted features. Decision tree learning was used as a classifier. Leave-one-out cross-validation estimated accuracy. Information gain was used to select features.	A task was posted on Mechanical Turk to allow participants to take the questionnaire. Tweets were collected from their public profiles.	200 tweets for each of 135 users	Depressive Symptom Inventory-Suicide Subscale Interpersonal Needs Questionnaire Acquired Capability for Suicide Scale	TW	English

First author, date, reference	Methodology	Machine language techniques	Data collection	Sampling	Survey	Site	Language of the study
Coppersmith, 2016 [31]	Datasets were preprocessed and URLs and user identifiers were replaced. Features were extracted from tweets. A classification model was trained and then tested.	Ngram techniques were used to extract information. Logistic regression was used to train a classifier.	Datasets were collected from Twitter by searching posts with statements about suicide attempts. An annotator hand verified each tweet.	554 users with suicide attempt statement	None	TW	English

First author, date, reference	Methodology	Machine language techniques	Data collection	Sampling	Survey	Site	Language of the study
Lv, 2015 [32]	This study was divided into 2 phases. The first was building the dictionary. Researchers identified initial words from the posts and 2 Chinese sentiment dictionaries (HowNet and National Taiwan University Sentiment Dictionary). A corpus-based method was used to expand the initial words. The second phase was testing the dictionary. The performance of the dictionary was tested at the level of posts and users. Classification models on the dictionary and	SVM models were built to make a 2-group classification. Simplified Chinese LIWC was used to analyze posts. Word2vec estimated the semantic similarity between different words.	To build the Chinese suicide dictionary, posts of users with and without suicidal ideation were searched from the site. For testing, an advertisement was posted on the site, and participants who agreed to engage completed a questionnaire and consented to download of their posts.	7908 words to build the dictionary; 788 participants in the testing phase	SPS[n]	Sina Weibo	Chinese

First author, date, reference	Methodology	Machine language techniques	Data collection	Sampling	Survey	Site	Language of the study
O'Dea, 2015 [33]	Researchers hand-coded tweets to determine the level of suicide-related concern. Machine classification models were developed. The accuracy of the classifiers was verified.	SVM and logistic regression algorithms were used to build machine classifiers. TF-IDF was used to weight the importance of target words in a document.	Tweets were collected through search APIs with a set of keywords.	14,701 suicide-related tweets	None	TW	English
Liu, 2015 [34]	Words were classified as positive or negative with LIWC. Correlation was estimated to evaluate the relationship between emotional expression and well-being.	LIWC was used to analyze posts. A multiple mediation model tested the effects of emotional expression on well-being.	Downloaded from myPersonality project.	134,087 posts downloaded from 1124 participants who granted access to their profiles on myPersonality	SWLS	FB	English

First author, date, reference	Methodology	Machine language techniques	Data collection	Sampling	Survey	Site	Language of the study
Burnap, 2015 [35]	Human annotators (crowdsourcing service) classified tweets into 7 categories. Features were extracted from the tweets. Classifiers were built to classify tweets into the same 7 categories.	TF-IDF identified words that can discriminate between suicidal ideation and nonsuicidal ideation classes. Stanford Log-linear Part-Of-Speech Tagger, WordNet Domains, SentiWordNet, ngram, and LIWC extracted textual features. Dimension reduction was achieved by PCA. SVM, decision trees, and naive Bayes were used to build classification models.	Posts from 4 suicide-related websites and Tumblr were collected to create a set of keywords to search for suicide-related tweets. The set of suicide-related keywords were used to search further tweets for analysis.	1000 tweets	None	TW	English

First author, date, reference	Methodology	Machine language techniques	Data collection	Sampling	Survey	Site	Language of the study
Park, 2015 [36]	Facebook features were correlated with CES-D[o] score.	Pearson correlation coefficient and simple linear regression analysis measured the relationship between CES-D scores and activities.	The researchers developed a Facebook app that allowed participants to take surveys and provide consent.	212 participants in large Korean universities	CES-D<br>BDI[p]	FB	Korean
Hu, 2015 [37]	Features were extracted. Classifiers on differences of observation time were developed. The models were verified.	WenXin, a Chinese text analysis program, was used to extract psychological and linguistic features. Greedy stepwise algorithm was used for feature selection. Logistic regression and linear regression were used to build classifiers.	Participants took a survey and provided consent. Participants' posts were downloaded.	10,102 participants who took the survey	CES-D	Sina Weibo	Chinese

First author, date, reference	Methodology	Machine language techniques	Data collection	Sampling	Survey	Site	Language of the study
Tsugawa, 2015 [38]	Features were extracted. Classifiers were built based on selected features.	MeCab, a Japanese morphological analyzer, was used. LDA was employed to find topics of each tweet. SVM built classifiers.	Participants took surveys and provided consent. Tweets were collected by a Twitter API.	A maximum of 3200 tweets collected from each of 209 participants	CES-D<br>BDI	TW	Japanese
Zhang, 2015 [39]	A predictive model was built and its accuracy was evaluated.	LIWC for a Chinese version extracted features. LDA extracted a topic for each post. Linear regression analysis with a stepwise selection algorithm was used to implement prediction models.	Participants completed the survey. Their posts were downloaded.	697 Weibo users	SPS	Sina Weibo	Chinese

First author, date, reference	Methodology	Machine language techniques	Data collection	Sampling	Survey	Site	Language of the study
Coppersmith, 2015 [40]	Age and sex were detected automatically. Features were extracted from tweets. The relationship between health conditions and language was assessed by Pearson correlation coefficient.	LIWC calculated attributes of language from tweets. Character ngram language models investigated character sequences and were trained as classifiers.	Tweets were searched and downloaded.	2013 users	None	TW	English
Preotiuc-Pietro, 2015 [41]	Several methods were used for feature extraction. Classifiers were built to determine which groups users belonged to, and their predictions were then verified.	Unigram, normalized pointwise mutual information, Word2vec, GloVe, and LDA were employed to extract features. Classification models were generated with logistic regression with elastic net regularization and SVM with a linear kernel.	Datasets were taken from Coppersmith et al [73].	327 users with depression, 246 users with PTSD[q], and a random sample of 572 users	None	TW	English

First author, date, reference	Methodology	Machine language techniques	Data collection	Sampling	Survey	Site	Language of the study
Mitchell, 2015 [42]	A human annotator selected tweets related to schizophrenia. Automatic tools extracted important features. Machine learning algorithms trained and tested data.	LIWC analyzed text content. LDA searched for topics in documents. Brown clustering was used to categorize words. Character ngram models represented the sequence of characters. Perplexity calculated the breadth of the used language. SVM and maximum entropy models built classifiers.	Datasets were searched and filtered by regular expressions.	174 users with schizophrenia, together with a random dataset	None	TW	English

First author, date, reference	Methodology	Machine language techniques	Data collection	Sampling	Survey	Site	Language of the study
Preotiuc-Pietro, 2015 [43]	Each user in the datasets was assigned age, sex, and personality with a set of tools. Each tweet was labelled in terms of emotional polarity and intensity. Features were extracted. Classifiers were built.	Ngram and pointwise mutual information extracted features. LIWC extracted textual features. Binary logistic regression with elastic net regularization was used to build classifiers.	Datasets were searched and filtered by regular expressions.	370 users with PTSD, 483 users with depression, and 1104 random users	None	TW	English
Pedersen, 2015 [44]	Ngrams were extracted from tweets to create decision lists. These lists were used to match ngrams from the tweets of each user. The match of ngrams and lists was calculated.	Ngrams were used to generate decision lists.	Datasets were obtained from Coppersmith et al [73].	327 users with depression, 246 users with PTSD, and a random sample of 572 users	None	TW	English

First author, date, reference	Methodology	Machine language techniques	Data collection	Sampling	Survey	Site	Language of the study
Resnik, 2015 [45]	Contents were analyzed with topic models. Their performance was tested.	LDA, supervised LDA, and supervised anchor model were used to find topics on tweets. The linear support vector regression model was the classification model.	Datasets were taken from a related study [73].	327 users with depression, 246 users with PTSD, and a random sample of 572 users	None	TW	English
Resnik, 2015 [46]	Features were extracted. Classifiers were tested by subsets of data.	Supervised LDA, supervised anchor model, and lexical TF-IDF extracted textual features. Classifiers were built with SVM with a linear kernel and RBF kernel.	Datasets were obtained from a related study [73].	327 users with depression, 246 users with PTSD, and a random sample of 572 users	None	TW	English

First author, date, reference	Methodology	Machine language techniques	Data collection	Sampling	Survey	Site	Language of the study
Durahim, 2015 [47]	Data were preprocessed to exclude tweets without location and users who tweeted <90 days in 2013. Each tweet was assigned a positive or negative polarity value. The results were verified with actual national data.	SentiStrength was used to measure the positive or negative polarity of each tweet.	Public tweets were collected using a Twitter API.	35 million tweets from >20,000 users	None	TW	Turkish
Guan, 2015 [48]	Features were extracted from posts and users' profiles. Two classifiers were trained and tested.	Simplified Chinese Micro-blog Word Count Dictionary, a Chinese version of LIWC, and TextMind extracted features. Logistic regression and random forest were used to train classifiers.	Researchers posted recruitment information on Sina Weibo. Participants took the survey and provided informed consent. Participants' posts were downloaded using APIs.	909 participants	SPS	Sina Weibo	Chinese

First author, date, reference	Methodology	Machine language techniques	Data collection	Sampling	Survey	Site	Language of the study
Landeiro Dos Reis, 2015 [49]	Features were extracted from a set of annotated tweets. These features were used to train classifiers.	Ngrams were used to create a list of words. LIWC extracted features. Logistic regression was used to train classifiers.	Tweets were searched and downloaded with keywords. Annotators labelled these tweets.	1161 users regularly exercising and 1161 control users	None	TW	English
De Choudhury, 2014 [50]	Feature extraction obtained sets of potential predictors. Predictive models were built based on the extracted features and then verified.	LIWC was used to investigate the contents of each tweet. Stepwise logistic regression with forward selection developed statistical models.	Advertisements were published on several media (eg, mailing lists, blogs, websites, Facebook posts, and tweets). Participants accessed the ads, and then completed the survey, providing informed consent to access their Facebook profiles.	578,220 posts from 165 participants	Patient Health Questionnaire-9	FB	English

First author, date, reference	Methodology	Machine language techniques	Data collection	Sampling	Survey	Site	Language of the study
Huang, 2014 [51]	Several tools were used to extract sets of features. Weka was employed to build a set of classifiers.	HowNet, a Chinese emotional lexicon, was used to conduct sentiment analysis. Ansj, a Chinese word segmentation tool, was used to segment words and ngrams, and to remove punctuation and stop words. SVM with an RBF kernel, naive Bayes, logistic regression, Weka J48 classifier, random forest, and sequential minimal optimization were used to build models.	Data were collected through the Sina API.	30,000 posts from 53 suicide ideation-related users and 600,000 posts from 1000 random users	None	Sina Weibo	Chinese

First author, date, reference	Methodology	Machine language techniques	Data collection	Sampling	Survey	Site	Language of the study
Wilson, 2014 [52]	Text analysis was performed and the results were compared across 2 groups. Each tweet was manually coded and assigned to a relevant group. The groups were compared in 10 linguistic features from LIWC.	LIWC was used to analyze content. An Analysis of variance was conducted on each linguistic feature.	Tweets were searched with keywords and downloaded.	13,279 depression-related tweets and 14,727 nondepression-related tweets	None	TW	English

First author, date, reference	Methodology	Machine language techniques	Data collection	Sampling	Survey	Site	Language of the study
Coppersmith, 2014 [53]	Classifiers were implemented based on machine learning techniques. Cross-validation was used to evaluate classification accuracy.	A unigram language model examined individual words. A character ngram language model examined a string of characters. LIWC extracted linguistic features. A log-linear regression model classified users.	Tweets related to PTSD were collected through search API and manually screened.	244 PTSD users and 5728 random users	None	TW	English

First author, date, reference	Methodology	Machine language techniques	Data collection	Sampling	Survey	Site	Language of the study
Kuang, 2014 [54]	LIWC and PERMA lexicons were translated to a Chinese corpus and extended by machine learning techniques. A subset of posts was manually coded. The annotated dataset was used to test the corpus. The corpus computed happiness scores through large-scale data.	Chinese word segmentation was performed. Pointwise mutual information measured similarity between each new word and the lexicon. Word2vec computed a distributed representation of each word to expand the corpus words.	Tweets were collected from the site with an API.	1.9 billion posts from 1.4 million users	None	Sina Weibo	Chinese

First author, date, reference	Methodology	Machine language techniques	Data collection	Sampling	Survey	Site	Language of the study
Hao, 2014 [55]	Features were extracted. A predictive model was developed and verified.	A Chinese version of LIWC was used to extract linguistic features. Regression algorithms, including stepwise regression, least absolute shrinkage and selection operator, multivariate adaptive regression splines, and support vector regression were used to build classifiers.	Participants received an invitation to take part in the project, and then completed surveys and provided consent to access their profiles. Posts were collected with an API.	1785 participants	Positive and Negative Affective Scale Psychological Well-Being Scale	Sina Weibo	Chinese

First author, date, reference	Methodology	Machine language techniques	Data collection	Sampling	Survey	Site	Language of the study
Prieto, 2014 [56]	The filtered posts were screened and tagged as a nonhealth-related condition or a health-related condition by human annotation. Classifiers were built to identify health conditions and the results were then tested.	SVM, naıve Bayes, decision trees, and k-nearest neighbor techniques were used to implement classifiers. Character bigram classified opposite word meanings. Bag of word extracted words from tweets and represented them. Word stem was used to find word roots and reduce each word to its root. Correlation-based feature selection, Pearson correlation, gain ratio, and relief methods were used to select features.	Tweets were collected using the Twitter search API and regular expressions.	5.8 million Spanish tweets and 7096 Portuguese tweets	None	TW	Spanish and Portuguese

First author, date, reference	Methodology	Machine language techniques	Data collection	Sampling	Survey	Site	Language of the study
Lin, 2014 [57]	Features, including text, image, and interactions, were extracted. A classifier was built. Other datasets were collected to test the model.	The Chinese version of LIWC was used to extract linguistic features. A convolutional neural network algorithm was used to learn and summarize relationships between extracted attributes of each tweet. Deep neural network implemented a classifier.	To build the models, tweets were collected by using Sina Weibo steaming APIs, and then filtered by phrases. To test the models, posts were collected from Sina Weibo, Tencent Weibo, and Twitter.	Posts of 23,304 users on Sina Weibo for building classifiers, and another 3 datasets from Sina Weibo, Tencent Weibo, and Twitter for testing models	None	Sina Weibo	Chinese
Schwartz, 2014 [58]	Feature extraction was performed. A classifier was constructed.	Ngram, LDA, and LIWC extracted textual features. PCA was used to reduce features. Linear regression was employed to build classifiers.	Datasets were downloaded from myPersonality project.	28,749 Facebook users	NEO Personality Inventory-Revised	FB	English

First author, date, reference	Methodology	Machine language techniques	Data collection	Sampling	Survey	Site	Language of the study
Coppersmith, 2014 [59]	Features were extracted. A classifier was built to distinguish each group.	Ngram and LIWC were used to extract textual features. A log-linear classifier was trained.	Tweets with diagnostic statements were searched and then all tweets from particular users were downloaded.	2.9 million tweets from 1238 users and 13.7 million tweets from 5728 users	None	TW	English
Homan, 2014 [60]	Tweets were annotated by novices and experts. Features were extracted from the annotated tweets, and then used to train models. The models were tested with a testing set.	LIWC captured linguistic features. Ngram extracted features from text and TF-IDF measured frequency of features. LDA captured a topic from each tweet. SVM was used to train classification models.	Tweets were collected with the search API.	2000 tweets from users in New York, NY	None	TW	English

First author, date, reference	Methodology	Machine language techniques	Data collection	Sampling	Survey	Site	Language of the study
Park, 2013 [61]	Spearman rank correlation coefficient, Mann-Whitney U test, and regression analysis were used to examine relationships between Facebook features and CES-D scores.	Spearman rank correlation coefficient and simple linear regression measured relationships between features and depression scores. Mann-Whitney U was used to estimate differences in features between depressed and nondepressed groups.	A Facebook app was developed. Participants took surveys and provided consent on the app.	55 participants	CES-D<br>BDI	FB	Korean

First author, date, reference	Methodology	Machine language techniques	Data collection	Sampling	Survey	Site	Language of the study
Wang, 2013 [62]	Features were extracted from posts. Sentence polarity was computed to estimate the intensity of sentiment. Detection models were based on Bayes networks, decision trees, and rules, and then 10-fold cross-validation was performed.	HowNet, a Chinese corpus and sentiment analysis program, was employed to recognize the polarity of each word. ICTCLAS, a Chinese word segmentation system, was used to segment work and identify the parts of speech of each word. Bayes networks, decision trees, and rules were used to build predictive models. Binary logistic regression analysis was performed for feature reduction.	Diagnoses were made with participant surveys and by psychologist interviews. Participants' posts were collected through the site API.	6013 posts from 180 depressed and nondepressed users	None	Sina Weibo	Chinese

First author, date, reference	Methodology	Machine language techniques	Data collection	Sampling	Survey	Site	Language of the study
Wang, 2013 [63]	A prediction model was built based on 3 different classifiers: (1) node features (only considers users with depression based on their posts), (2) node features and tie strength (computes a label of each node and relationships of the node to neighbor nodes), and (3) node features and interaction content (classifies the presence of depression based on the label of a node and interactions produced by the node). The model was trained and verified.	A decision tree technique was used to build models. Sentiment analysis was performed to label each user.	Diagnoses were made with participant surveys and by psychologist interviews. Participants' posts were collected through the site API.	27,518 posts and 17,596 links from 100 training users	None	Sina Weibo	Chinese

First author, date, reference	Methodology	Machine language techniques	Data collection	Sampling	Survey	Site	Language of the study
Tsugawa, 2013 [64]	Word frequencies were estimated. Regression models estimated depressive tendencies and compared these estimations with self-reported scores in the testing group.	MeCab, a tool for Japanese morphological analysis, was used to generate frequencies of words. A regression model with stepwise selection was used to build predictive models.	Participants were recruited in the authors' laboratories. Participants completed a survey and their tweets were collected with the Twitter API.	3200 tweets for each of 50 participants	Zung Self-Rating Depression Scale	TW	Japanese
De Choudhury, 2013 [65]	Features were extracted and then used as vectors to build predictive models.	LIWC and affective norms for English words were performed to compute emotional state and linguistic style. SVM with an RBF kernel was used to build a classification model. PCA was performed to reduce feature redundancy.	Search terms were taken from newspaper announcements of births. The Twitter Firehose stream was used to collect and filter tweets with these search terms. Crowdsourced workers identified tweets from users who were likely to be new mothers.	376 users with 36,948 posts during the prenatal period and 40,426 posts during the postpartum period	None	TW	English

First author, date, reference	Methodology	Machine language techniques	Data collection	Sampling	Survey	Site	Language of the study
De Choudhury, 2013 [66]	Sets of features were extracted. The feature sets were used to train classifiers. These classifiers were used to label daily public posts and then compute depression levels. The predictions were compared with the actual results.	LIWC and an Affective Norms for English Words lexicon were used to extract linguistic styles and measure emotions. Ngrams were used to detect informal language use. SVM with an RBF kernel was used to implement prediction models. PCA was used to reduce feature redundancy.	Mechanical Turk volunteers took a questionnaire and granted access to their public posts.	69,514 posts from 489 users to build models	CES-D	TW	English

First author, date, reference	Methodology	Machine language techniques	Data collection	Sampling	Survey	Site	Language of the study
De Choudhury, 2013 [67]	A depression lexicon was generated and features were extracted from Twitter data. A classifier was developed and tested.	LIWC was used to measure emotions and extract linguistic styles. Pointwise mutual information, log likelihood ratio, and TF-IDF generated a depression lexicon. SVM with an RBF kernel was built as a classifier. PCA was used to reduce overfitting.	Volunteers completed a survey on Mechanical Turk and granted access to their public posts.	2 million posts from 476 participants	CES-D<br>BDI	TW	English

First author, date, reference	Methodology	Machine language techniques	Data collection	Sampling	Survey	Site	Language of the study
Schwartz, 2013 [68]	Geolocation was extracted from tweets. Extracted features were used to train a model and then the accuracy of the model was tested. Predictions were aggregated by country, and then matched to locations to map life satisfaction. The aggregated results were compared with actual collected statistics.	LIWC and PERMA were used to extract linguistic features. LDA was used to extract topics. Least absolute shrinkage and selection operator linear regression technique was used to train a predictive model.	Tweets were collected via the Twitter Garden Hose.	Tweets containing >30,000 words from each of 1293 US counties	None	TW	English

First author, date, reference	Methodology	Machine language techniques	Data collection	Sampling	Survey	Site	Language of the study
Hao, 2013 [69]	Two classifiers were built. First, a lexicon classifier was built based on participants' words. Second, a classification model was trained with linguistic and behavioral features. Then, both models were tested.	SVM, naive Bayes, and neural network techniques were used to train classifiers. Simplified Chinese LIWC was used to extract features.	Researchers invited participants to log in to their app to complete a survey. Social network data were downloaded using APIs.	448 participants	Symptom Checklist-90-Revised	Sina Weibo	Chinese
Jamison-Powell, 2012 [70]	Content analysis was performed to detect characteristics of tweets. Statistical analysis was used to find significant differences in the characteristics. Human coding was conducted to identify a theme for each tweet.	LIWC performed content analysis.	Tweets with the #insomnia hashtag were collected with the Twitter search API. Random tweets were downloaded with the streaming API.	18,901 tweets with #insomnia and 17,532 tweets from a nonspecific sample	None	TW	English

First author, date, reference	Methodology	Machine language techniques	Data collection	Sampling	Survey	Site	Language of the study
Bollen, 2011 [71]	Subjective well-being was estimated from the textual content of tweets. Social network analysis was performed to find connections between users. Distribution, pairwise assortativity, and neighborhood assortativity were calculated to find correlations in the graph.	OpinionFinder, a sentiment analysis tool, was employed to analyze emotional content.	Tweets and follower network graphs of each user were collected with the Twitter API.	129 million tweets from >4 million users	None	TW	English

[a]LIWC: linguistic inquiry and word count.

[b]SVM: support vector machine.

[c]RBF: radial basis function.

[d]TW: Twitter.

[e]TF-IDF: term frequency-inverse document frequency.

[f]GLoVe: global vectors for word representation.

[g]API: application programming interface.

[h]PERMA: positive emotions, engagement, relationships, meaning, and accomplishment.

[i]SWLS: Satisfaction With Life Scale.

[j]LDA: latent Dirichlet allocation.

[k]PCA: principal component analysis.

[l]FB: Facebook.

[m]IG: Instagram.

[n]SPS: Suicide Probability Scale.

[o]CES-D: Center for Epidemiologic Studies Depression Scale.

[p]BDI: Beck Depression Inventory.

[q]PTSD: posttraumatic stress disorder.
